# Understanding the Temporal Evolution of Isoprene Organosulfates Using a Novel Hydrophilic Interaction Chromatography Method

**DOI:** 10.1021/acsestair.5c00460

**Published:** 2026-07-07

**Authors:** Ping Liu, Daniel J. Bryant, Carys Williams, Rhianna L. Evans, James R. Hopkins, Sri H. Budisulistiorini, Michael Flynn, Nathan A. I. Watson, James D. Allan, W. Joe F. Acton, William. J. Bloss, Connor Prior, William Unsworth, Xiang Ding, Andrew R. Rickard, Jacqueline F. Hamilton

**Affiliations:** † State Key Laboratory of Advanced Environmental Technology, 152530 Guangzhou Institute of Geochemistry, Chinese Academy of Sciences, Guangzhou 510640, China; ‡ Wolfson Atmospheric Chemistry Laboratories, Department of Chemistry, 8748 University of York, York YO10 5DD, U.K.; § National Centre for Atmospheric Science, University of York, Heslington, York YO10 5DD, U.K.; ∥ Department of Earth and Environmental Sciences, 5292 University of Manchester, Manchester M13 9PL, U.K.; ⊥ National Centre for Atmospheric Science, University of Manchester, Manchester M13 9PL, U.K.; # School of Geography Earth and Environmental Science, University of Birmingham, Edgbaston, Birmingham B15 2TT, U.K.; ∇ Department of Chemistry, University of York, Heslington, York YO10 5DD, U.K.; ○ Guangdong-Hong Kong-Macao Joint Laboratory for Environmental Pollution and Control, Guangzhou Institute of Geochemistry, Chinese Academy of Science, Guangzhou 510640, China; ◆ Now at School of Chemistry, University of Leeds, Leeds LS2 9JT, U.K.

**Keywords:** secondary organic aerosol, isoprene, organosulfate, orbitrap mass spectrometry, HILIC

## Abstract

Organosulfates (OSs) form a significant component of secondary organic aerosol (SOA) in the atmosphere. Previously, their measurement using reversed-phase liquid chromatography–high-resolution mass spectrometry (RPLC–HRMS) has led to poor retention and significant matrix effects. Hydrophilic interaction liquid chromatography (HILIC) provides improved retention of polar OSs, such as those formed from isoprene oxidation, and reduced matrix effects. Studies employing HILIC predominantly use gradient elution to separate OSs with varying polarities. HILIC, however, is very sensitive to changes in the water layer within the column, which facilitates separation, meaning the column needs to be thoroughly equilibrated before each injection, significantly increasing analysis time, cost, and solvent waste. The work presented here describes the development of an isocratic HILIC method to analyze OSs in complex ambient PM_2.5_ samples. This new method significantly reduces solvent usage and analysis time, which is vital for the analysis of large quantities of samples. The method was first validated with synthesized isoprene-derived OS tracers (iOS) and commercially available standards, then applied in the iOS analysis of filter samples collected during the summertime in Manchester, England. Eight filter samples were taken each day, allowing the temporal evolution of iOS tracers to be elucidated. For 336 ambient samples analyzed in this work, the adoption of the isocratic method resulted in a total time saving of 61 h and reduced organic solvent usage by 1.68 L. The targeted analysis of iOS was quantified using authentic standards. Five 2-methyltetrol OS isomers were quantified (C_5_H_12_O_7_S; MTOS_1–4, 6) averaging 0.36, 0.11, 1.25, 2.12, and 0.17 ng m^–3^, respectively. The average concentration of total 2-methyltetrol OS was 3.99 ng m^–3^, and 2-methylglyceric acid OS (C_4_H_8_O_7_S: MGOS) was 1.07 ng m^–3^.

## Graphical abstract

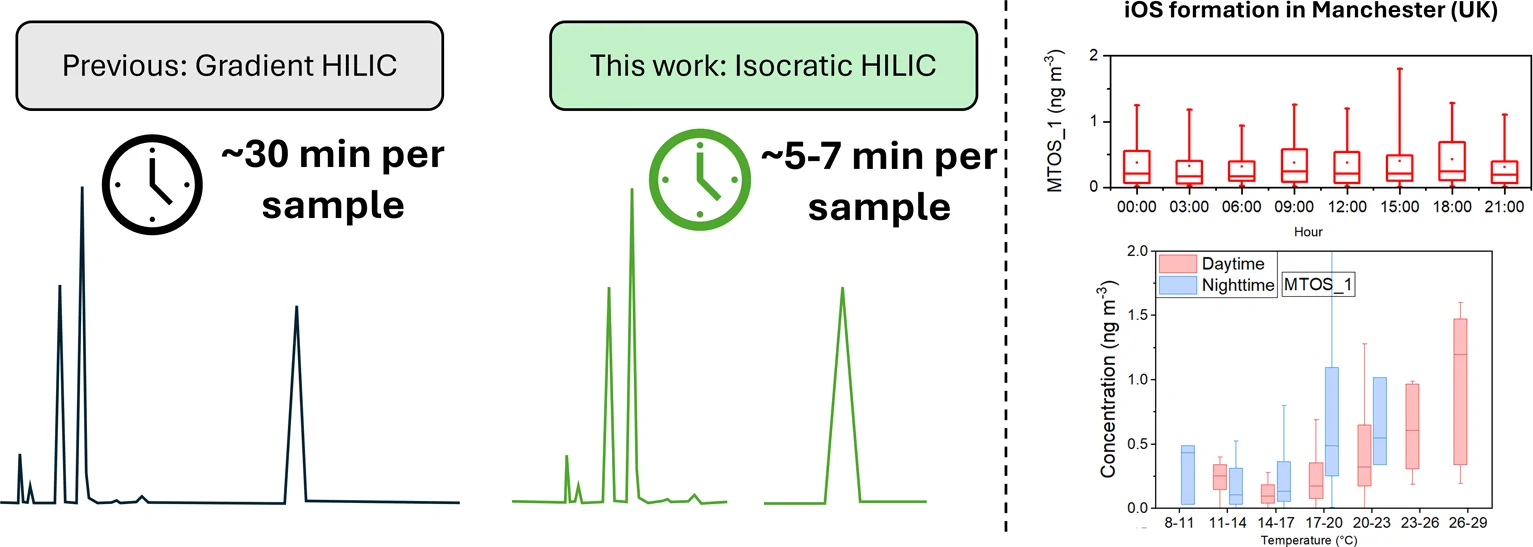

## 1 Introduction

Organosulfates (OSs) are ubiquitous constituents of atmospheric aerosol particles and have commonly been used as secondary organic aerosol (SOA) markers.^1–12^ In addition, Romero and Oehme first reported the presence of OS in aerosols, tentatively proposing that they were a component of humic-like substances (HULIS) in fine particulate matter.^13^ Isoprene is globally the most abundant nonmethane biogenic hydrocarbon precursor of SOA, with isoprene-derived OSs (iOSs) being observed in organic aerosol at total mean iOS concentrations of >1000 ng m^–3^.^14–16^ Generally, they are assumed to form via biogenic-anthropogenic interactions through multiphase reactions between isoprene oxidation products and acidic sulfate particles.^3,10,11,17^ However, studies also highlight that OSs originate from anthropogenic precursors.^18–22^ Thus, iOSs can significantly affect atmospheric chemistry due to these elevated concentrations.

The most common approach to measuring OSs in atmospheric particles is the analysis of filter extracts by liquid chromatography coupled to mass spectrometry with electrospray ionization (LC-ESI-MS).^11,19,23^ Because of the acidic proton of sulfate functionalities, derivatization is not required, and high ionization efficiencies in negative polarity are observed, giving deprotonated molecular ions ([M-H]^−^). Studies investigating OSs often use reverse-phase liquid chromatography (RPLC) for the detection and quantification of OSs in ambient particles, for example, SOA derived from monoterpenes.^6,12,18,24–27^ However, for smaller and more polar OSs such as iOSs, RPLC cannot effectively retain the analytes or provide isomeric separation. Hydrophilic interaction liquid chromatography (HILIC) is a viable alternative and was recently shown to be capable of resolving polar OSs effectively.^15,16,28–30^

Previous HILIC methods use a gradient elution method such as RPLC, which improves the separation of complex samples. However, there are associated disadvantages of using a gradient HILIC method when targeting specific analytes. HILIC was introduced to address the need for a separation method that would retain highly polar molecules such as peptides and nucleic acids.^31^ Separation of analytes is controlled by the differential distribution of analyte molecules between the organic-rich mobile phase and the aqueous layer adsorbed onto the hydrophilic stationary phase.^32^ A more polar/hydrophilic molecule will have its partitioning equilibrium shifted toward the water layer on the stationary phase and, hence, will be more strongly retained in the column. During a gradient method, the water layer thickness changes, so before a subsequent sample injection, the water layer thickness needs to be reestablished. Failure to incorporate column equilibration into the methodology may result in the incomplete reestablishment of the water layer on the particle surface, consequently causing irreproducibility.^33^ The need to re-equilibrate the column after each sample results in long run times and high solvent usage, especially with large numbers of samples.

In this paper, we present a new isocratic method to detect specific iOS tracers, such as 2-methyltetrol organosulfates (MTOSs) and 2-methylglyceric acid organosulfate (MGOS), resulting in higher throughput and reduced solvent usage. This new methodology was then applied to determine the diel concentration profiles of iOS at the Manchester Air Quality Supersite, located in a suburban area of the city of Manchester in the UK.

## 2 Experimental Section

### 2.1 Field Sampling

PM_2.5_ filter samples were collected at the Manchester Air Quality Supersite (MAQS; WMO Integrated Global Observing System (WIGOS) i.d.: 0-20008-0-MCR) during the summer Observation System for Clean Air (OSCA) field campaign, from the 8th of June to the 20th of July in 2021. MAQS is an urban background site situated at the Firs Botanical Gardens on the University of Manchester’s Fallowfield Campus. Eight filter samples were collected per day (00:00–03:00, 03:00–06:00, 06:00–09:00, 09:00–12:00, 12:00–15:00, 15:00–18:00, 18:00–21:00, 21:00–00:00) using a Digitel DHA-80 high-volume sampler on quartz filters (circle diameter: 150 mm, prebaked for 5 h at 500 °C), at a flow rate of 0.5 m^3^ min^–1^. Field blanks were taken on the 8th of June. A prebaked blank filter was placed into the high-volume sampler without turning the sampler on for 10 min. After which, the filter was removed and handled in the same way as the PM_2.5_ samples. After sampling, the filter samples were immediately wrapped in the same prebaked aluminum foil and sealed in an airtight bag before being stored at −20 °C until extraction in 2023.

A range of supporting data, including gas (isoprene (ppb), NO_2_ (ppb), NO (ppb), O_3_ (ppb)), aerosol (PM_2.5_ (μg m^–3^), SO_4_ (μg m^–3^)), and meteorological data (temperature (°C), relative humidity (RH, %)), are available at https://data.ceda.ac.uk/badc/osca/data/manchester. Isoprene was measured via a Proton Transfer Reaction-Quadrupole ion guide Time-of-Flight-Mass Spectrometer (PTR-QiTOF-MS; Ionicon Analytik, Innsbruck) installed in an air-conditioned container at the MAQS. The instrument sampled from a height of 2.5 m via a heated inlet with a flow rate of 100 sccm. The instrument was operated with an *E*/*N* ratio (where *E* is the electric field strength, and *N* is the buffer gas density) of 120 Td. The PTR-QiTOF-MS was calibrated weekly using a multicomponent gas standard, containing isoprene (Apel-Riemer Environmental, Inc., Miami), dynamically diluted in zero air.

### 2.2 Extraction and Preparation

The procedure described by Hettiyadura et al.,^28^ was followed with slight modifications. A circular section with a diameter of 47 mm was excised from the quartz membrane using a stainless steel cutter, which was thoroughly cleaned between samples using methanol. This section was then cut into roughly 1 cm^2^ pieces and stored in a 20 mL glass vial. Next, 8 mL of chromatographic-grade MeOH (Optima, Fisher Chemical) was added to the sample and sonicated for 45 min. Ice packs were used to keep the bath temperature below room temperature, with the water swapped midway through. Using a 5 mL plastic syringe (Injekt, 4606051 V), the solvent extract was then pushed through a 0.22 μm filter (Millipore) into another sample vial. An additional 2 mL (2 × 1 mL) of MeOH was added to the filter sample and then extracted through the filter to give a combined extract of ∼10 mL. This extract was then reduced to dryness using a Genevac miVac Centrifugal Concentrator. The dry sample was then reconstituted in 95:5 ACN (LiChrosolv, Merck KGaA): H_2_O for analysis.

### 2.3 HILIC Method

Analysis was conducted utilizing an Ultimate 3000 UHPLC system (Thermo Scientific, USA) coupled with a Q Exactive Orbitrap MS (Thermo Fisher Scientific, USA), employing data-dependent tandem mass spectrometry (ddMS^2^) with a heated electrospray ionization source (HESI). The separation was achieved on a Waters ACQUITY UPLC BEH Amide column (2.1 × 100 mm, 1.7 μm particle size, Waters) using an isocratic elution method, with the column temperature maintained at 45 °C. For the targeted analysis of MTOS, methyl sulfate, and ethyl sulfate, a mobile phase consisting of 100% mobile phase B (95:5 ACN:water, pH 9) was employed,^28,30^ and the elution was held constant for 5–7 min at a flow rate of 0.5 mL min^–1^. For the targeted analysis of MGOS, glycolic acid sulfate, and lactic acid sulfate, an isocratic method utilizing 80% mobile phase B and 20% mobile phase A (water, pH 9) was employed, and this composition was held constant for 5 min at a flow rate of 0.5 mL min^–1^.

### 2.4 OS Standards

In this experiment, we used a total of six OS standards, two of which were provided by the Surratt group (MTOS, (purity: 69.8%), MGOS, (83.7%) (Cui et al., 2018; Rattanavaraha et al., 2016)). Two were synthesized in-house (glycolic acid sulfate (30.6%), lactic acid sulfate (59.8%), and two were purchased commercially (methyl sulfate (98%) and ethyl sulfate (>98%).

Glycolic acid (1 g, 13.15 mmol) was dissolved in ethyl acetate (33 mL) and cooled to −78 °C using acetone and dry ice. *N,N*-Diisopropylethylamine (2.63 mL, 15.12 mmol, 1.15 equiv) was then added in one portion. After 15 min of stirring at −78 °C, chlorosulfonic acid (0.96 mL, 1.68 g, 1.10 equiv) was added dropwise over 10 min. The reaction mixture was allowed to equilibrate to room temperature over 16 h. The reaction mixture was concentrated under reduced pressure to yield a viscous, colorless oil (2.5 g).

Lactic acid (1.18 g) was dissolved in ethyl acetate (33 mL) and cooled to −78 °C using acetone and dry ice. *N,N*-Diisopropylethylamine (2.63 mL, 15.12 mmol, 1.15 equiv) was then added in one portion. After 15 min of stirring at −78 °C, chlorosulfonic acid (0.96 mL, 1.68 g, 1.10 equiv) was added dropwise over 10 min. The reaction mixture was allowed to equilibrate to room temperature over 16 h. The reaction mixture was concentrated under reduced pressure to yield a viscous red oil (3.5 g).

In order to determine the purity of the glycolic acid sulfate and lactic acid sulfate standards, quantitative nuclear magnetic resonance (^1^H NMR) spectroscopy analysis was employed, relying on the comparison of integrated spin peak intensities.^34,35^ This method quantifies purity by correlating integrals with molar concentrations of defined protons. Quantitative analysis involves several parameters: Pa, Ia, Na, Ma, and Wa represent the purity, integral value of a specific chemical shift, number of protons in the signal, molar mass, and weighed mass of the material under investigation, respectively. Correspondingly, Ps, Is, Ns, Ms, and Ws denote the purity, integral value, number of protons, molar mass, and weighed mass of the standard, respectively; see eq 1.

1 $$P_{a}=\frac{I_{a}}{I_{s}}\cdot \frac{N_{s}}{N_{a}}\cdot \frac{M_{a}}{M_{s}}\cdot \frac{W_{s}}{W_{a}}\cdot P_{s}$$

Prior to computation, knowledge of proton chemical shift values within the compound’s constituent groups is essential. A suitable peak is then selected for purity determination. Ultimately, through calculation, the purity of glycolic acid sulfate was determined to be 30.6%, while lactic acid sulfate exhibited a purity of 59.8%.

## 3 Results and Discussion

### 3.1 Standard Curve

In previous studies, owing to the lack of authentic standards, the quantitative errors for MTOS and MGOS could exceed 200%.^26^ Even when Liu et al. employed a new quantification method and substituted reference and internal standards, the quantitative errors remained as high as 75%.^30^ Figure S1 presents the standards’ calibration curves used in this study. 7-point calibration curves were constructed for all standards at concentrations of 0.0025, 0.005, 0.01, 0.025, 0.05, 0.1, and 0.25 μg mL^–1^, yielding high correlation coefficients (*r* ^2^) (Figure S1). For MTOS, where multiple isomers were detected in the ambient samples, peak 3 (MTOS_3) was employed for quantifying peaks 1–3 (RTs: 1.01, 1.21, 1.78 min), while peak 4 was employed for peaks 4 and 5 with retention times of 2.22 and 3.20 min. This experiment demonstrates the excellent linearity of the method for these standards. The method exhibits higher sensitivity toward compounds eluting via the 100% B method compared to eluents from the 20% A method, due to the higher volatility of the mobile phase mixture, which ionization.

### 3.2 Limit of Detection and Limit of Quantification; Relative Standard Deviations of the MS Method

To ensure the effectiveness of this method in monitoring the target compounds in ambient samples, the limit of detection (LOD) and limit of quantification (LOQ) were determined for the available standards. LOD can be calculated based on the standard deviation of the response (Sy) of the curve and the slope of the calibration curve (S) at levels approximating the LOD were established at the 95% confidence interval, calculated as formula: LOD = 3­(Sy/S). The Sy of the response can be determined based on the standard deviation of *y*-intercepts of regression lines. In this experiment, ambient air was sampled onto filters for 3 h, resulting in a sampling volume of 90 m^3^. Considering the entire analysis process, the method detection limits (LOQs) for these compounds were determined, calculated following: $\mathrm{LOQ}s=\mathrm{LODs}\times \frac{V_{1}}{V_{2}}$ ,^30^ the solution volume in the vial for analysis (*V*_1_) was 500 μL, and the air volume corresponding to the aliquot of filter analyzed (*V*_2_) was 28.2 m^3^.

The LOD and LOQ for each standard are presented in Table S1. Among the various standard samples analyzed, for MTOS_3 and MTOS_4, the LODs were 0.005 μg mL^–1^ and 0.004 μg mL^–1^, and the LOQs were 0.095 ng m^–3^ and 0.084 ng m^–3^, respectively. For MGOS, the LOD and LOQ were 0.007 μg mL^–1^ and 0.126 ng m^–3^, respectively. Notably, lactic acid sulfate (LAS) exhibited the highest LOQ at 0.449 ng m^–3^.

Furthermore, the stability of the instrument was assessed by conducting 10 parallel measurements of a 0.1 μg mL^–1^ standard. The Table S1 results indicated that the relative standard deviations (RSDs) for these standards did not exceed 6.48%, demonstrating minimal relative error and highlighting the reproducibility of the experiment. This suggests that the experiment is conducive to the detection of OSs. The stability of the analysis process ensures the reliability of the obtained results.

### 3.3 Matrix Effects

Matrix effects were assessed by comparing the signal intensity of the standard in a blank solvent matrix with that in the ambient aerosol matrix. These effects can manifest as either a decrease (ion suppression) or an increase (ion enhancement) in signal response, resulting from changes in the ionization efficiency of analytes in the presence of coeluting compounds.^36^ Identifying matrix effects is crucial for ensuring the production of reliable analytical results. We evaluated the matrix effect of ambient samples on the signal response of the four standards used for quantification. A 10 μL mixture containing MTOS, methyl sulfate, and ethyl sulfate at 0.1 μg mL^–1^ was spiked into either 100 μL of ambient filter sample extract or 100 μL of blank 95:5 (ACN:H_2_O) solvent (totaling 17 spiked ambient samples). The samples were then analyzed as described above.

Matrix effect factors were calculated by subtracting the areas of compounds already present in the ambient sample from the compound in the spiked ambient samples and then dividing by the areas in the blank matrix. A 100% value indicates no matrix effect. Table S2 presents the percentages across 17 ambient samples collected during the campaigns, which encompassed a mixture of high and low PM_2.5_ concentrations throughout different times of the day, see Table S3 for details. MTOS exhibited a significant matrix effect, with an average ratio of 47.30 ± 14.22%, suggesting a 53% suppression in signal response. In contrast, methyl sulfate and ethyl sulfate showed minimal matrix suppression, with averages of 72.5 ± 16.7% and 78.9 ± 12.7%, respectively.

## 4 Ambient Summertime Observations in Manchester

### 4.1 Meteorology and Anthropogenic Pollutants

Figure S2 displays the time series and diurnal variations of measured pollutants, including temperature, relative humidity (RH), PM_2.5_, NO_2_, NO, O_3_, SO_4_, isoprene, and boundary layer height (BLH) at the MAQS between 8th June and 20th July in 2021, with an average time of 3 h. Table S4 summarizes the maximum, minimum, and mean values of this data for the campaign period. Throughout the observation period, the average temperature was 17.0 ± 3.5 °C, up to a maximum of 28 °C. The average concentrations of NO_2_ and NO were 6.45 ± 3.80 ppb and 0.95 ± 1.02 ppb, respectively, representing a relatively low-NOx urban background environment. NO peaks in the early morning (6 a.m.), likely linked to a low boundary layer and increasing local traffic emissions. The highest NO_2_ concentrations are observed at night, with a minimum in the mid-afternoon, likely due to dilution effects and photochemical degradation. The concentration of O_3_ was relatively stable throughout the observation period, with an average concentration of 25.42 ± 9.71 ppb. The average concentration of PM_2.5_ was 6.41 ± 3.50 μg m^–3^ with little diurnal variation, with only a slight increase during nighttime hours. This pattern indicates that local sources are not the primary driver of PM_2.5_ levels in Manchester and that regional contributions likely play a more significant role.

Isoprene concentrations in Manchester were extremely low, with an average concentration of 0.038 ± 0.033 ppb and a maximum of 0.8 ppb. Isoprene concentrations at Manchester are therefore significantly lower than those observed in London, Shenzhen, and Hongkong.^37–39^ The average diurnal profile indicates that isoprene mixing ratios are higher during the day, as expected due to biogenic emissions. Figure 1 shows the relationship between isoprene and ambient temperature, where isoprene levels increase exponentially with temperature at daytime.^40–42^ The correlation between isoprene and daytime temperature is strong (Daytime: *r* ^2^ = 0.8449), while there is no correlation at night. The sustained nocturnal levels of isoprene (Figure S2) are potentially due to unreacted isoprene left over from the day and/or increased anthropogenic emissions from traffic-related sources.^37^

**Figure 1:**
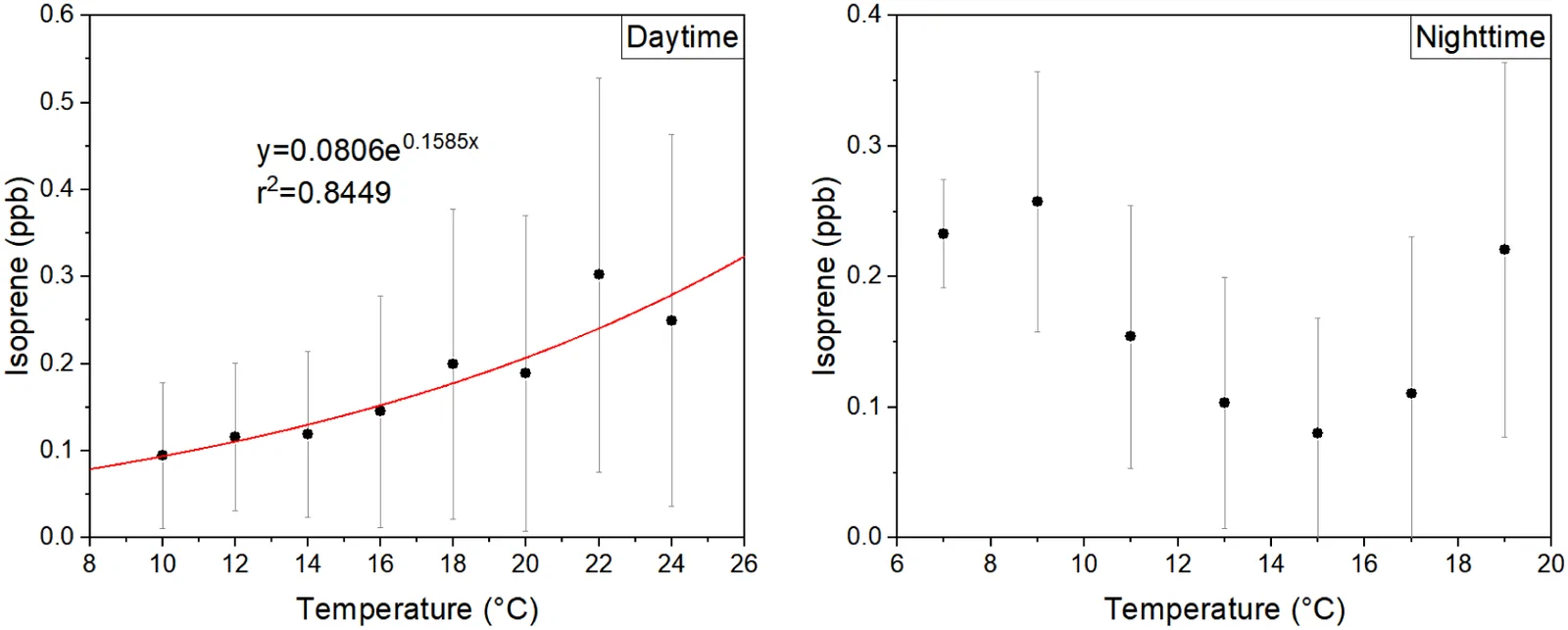
Correlation plots between isoprene and temperature. Daytime: 04:30–21:30, nighttime: 21:30–04:30.

### 4.2 Isoprene-Derived OSs

Figure S3 shows a simplified reaction scheme for the formation of iOSs, under varying atmospheric conditions. Isoprene epoxydiols (IEPOX) and hydroxymethyl-methyl-α-lactone (HMML) serve as key reactive intermediates in the oxidation of isoprene, prevailing under low-NO and high-NO conditions, respectively. Upon reactive uptake by acidic particles, IEPOX and HMML react with sulfate to yield MTOS and MGOS, respectively.^17,26,43^

However, recent work has shown that IEPOX can be formed via “high-NO” reaction pathways via the oxidation of isoprene hydroxynitrates (IHN) (Wang et al., 2025).^50^ In addition to formation via HMML, MGOS has also been detected as a heterogeneous OH oxidation product of MTOS and in the dilute aqueous-phase OH oxidation of MTOS;^44,45^ therefore, MGOS has multiple formation routes that can also involve “low-NO” reaction pathways.

In this work, for the analysis of MTOS, a mobile phase consisting of 100% mobile phase B (95:5 ACN:water, pH 9) was employed. For the analysis of MGOS, an isocratic method utilizing 80% mobile phase B (ACN) and 20% mobile phase A (water, pH 9) was employed. Cui et al. assigned six peaks observed in the MTOS (*m*/*z* 215) chromatogram to their respective isomeric structures,^16^ as shown in Figure S4. The first two peaks (A) are believed to be diastereomers of the compound 3-methyltetrol sulfate (peaks 1 and 2), with peaks 3 and 4 (B) consisting of 2-methyltetrol sulfate isomers. The final two peaks (C) are assigned to primary OSs derived from δ-IEPOX. Due to the higher abundance of the β-IEPOX isomer compared with the δ-IEPOX isomer, the last two peaks corresponding to the primary sulfates derived from δ-IEPOX only, which are lower in concentration when compared to the first four isomers. Peaks 1 and 3 are thought to be formed from trans-β-IEPOX, with peaks 2 and 4 suggested to be formed from cis-β-IEPOX,^46^ although there may also be contributions from the δ-IEPOX species to the first 4 peaks.^16^

In this work, five MTOS isomers (MTOS_1–4, 6) were observed in the Manchester samples. Figure S5 shows a box plot for the observed isomeric MTOS and MGOS’s concentrations, with the respective time series across the measurement period shown in Figure 2. In general, MTOS_4 had the highest mean concentration of 2.1 ± 3.5 ng m^–3^ and a maximum of 29.7 ng m^–3^. The next most abundant was MTOS_3 with a mean concentration of 1.25 ± 1.99 ng m^–3^, followed by MGOS (1.1 ± 1.6 ng m^–3^), while MTOS_1, and MTOS_2 had the lowest concentrations and could not be detected in some samples. The PM_2.5_ at the Manchester site had higher levels of total MTOS compared to MGOS (Figure S6). Previous studies have revealed that in the Pearl River Delta, China, even under high-NO pollution conditions, the concentration of IEPOX-derived MTOS remains higher than that of HMML-derived MGOS.^47^ Therefore, the relative ratio of these species is not only related to the levels of NO; other factors may influence the HMML production pathway, even under highly polluted conditions. Figure S7 shows correlation plots (R, corPlot, Openair) containing all iOS tracers observed during the measurement campaign and highlights the strong correlations observed between the iOSs. In the campaign, MTOS_3 and MTOS_4 have the strongest correlation (*R* = 0.96), indicating that they may have formed via the same chemical pathway. MTOS_2 and MTOS_6 also have a strong correlation (*R* = 0.92) indicating that they might have same source, similar formation pathways, and similar loss rates.

**Figure 2:**
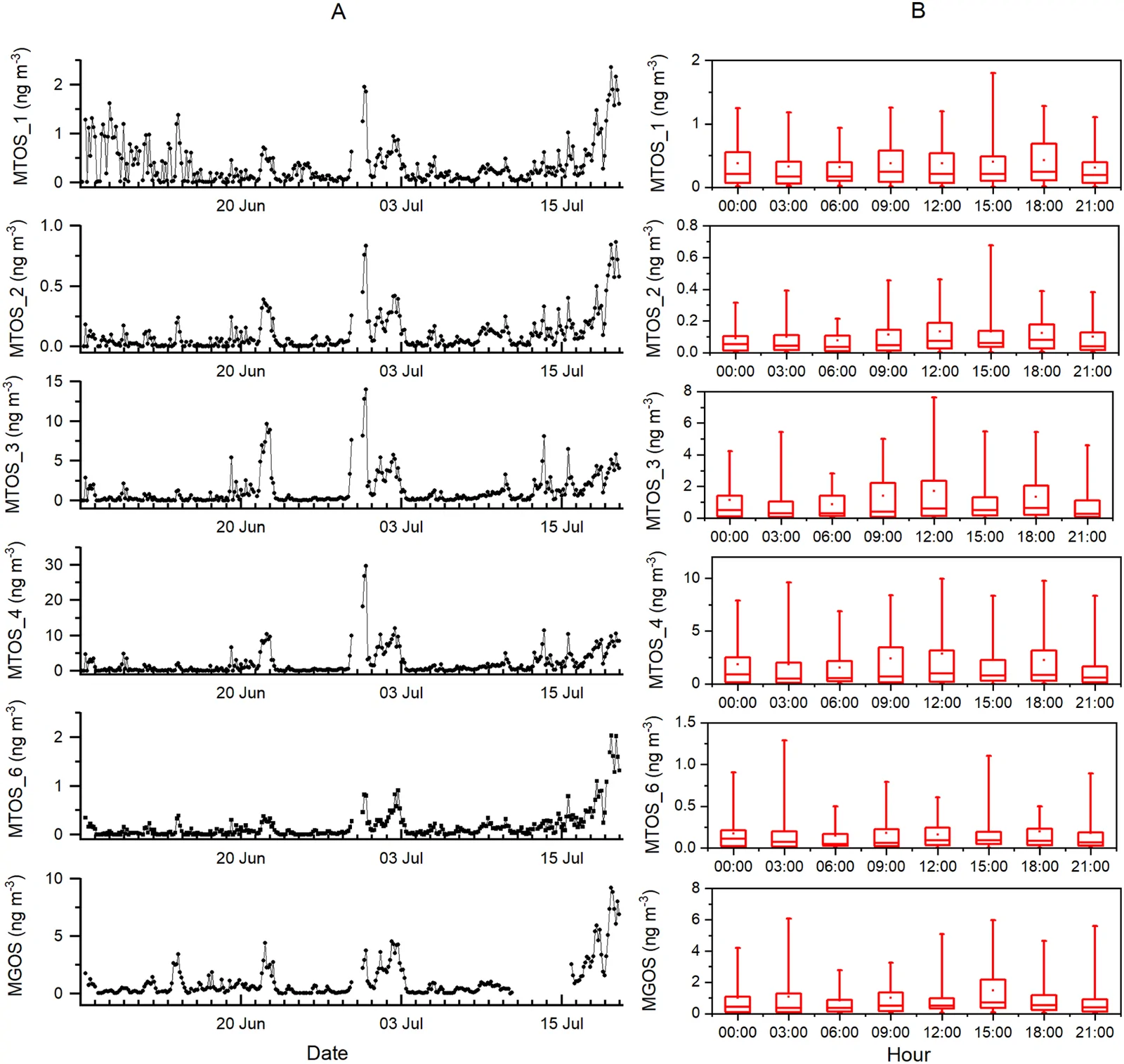
Time series and diurnal variation of MTOS (ng m^–3^) and MGOS (ng m^–3^) concentrations at the Manchester site between 08 June and 20 July.

The plot shown in Figure 2B gives the average diurnal variations of the observed MTOS isomers and the MGOS. The levels of MTOS_3 and MTOS_4 are relatively stable throughout the day, with two slightly elevated periods, one at night (03:00), possibly owing to the lower BL, and the second elevated period at about midday (12:00), indicating photochemical formation. The peaks of MTOS_1 and MTOS_2 appear at 15:00, which is also a period of relatively active photochemistry. There are two peak periods for MTOS_6 and MGOS, one at night which is the same as MTOS_3 and MTOS_4, and the second elevated period at 15:00, which may indicate the photochemical reactions.

In previous studies, the ratio of the average concentration of the commonly used iOS tracers from the low-NO versus the high-NO pathways (2-MTOS/2-MGOS) was reported as follows: Guangzhou summer (4.85), Guangzhou winter (0.84), Beijing (0.51), Hefei (0.42), Kunming (0.96), Delhi summer (1.97), and Delhi fall (0.37).^18,26,27^ In this study, a comparison of low-NO and high-NO iOS formation pathways revealed that the low-NO pathway was important both during the day and at night, with the high-NO pathway increasing significantly during the daytime (Figure S6), indicating that low-NO oxidation chemistry plays a significant role in iSOA formation at the Manchester site.

The high-resolution automated filter sampling system and the isocratic method employed in this study have allowed us to investigate the temporal evolution of MTOS isomers and MGOS for the first time. Across the whole campaign, low concentrations were observed during most days, but the concentrations increased significantly during three periods: 20th–21st June, 30th June to 2nd July, and 17th to 20th July (Figure 2). In order to investigate the relative contributions of local and longer-range transport of iOS to the site, the wind direction-dependent concentrations of iOSs are shown in the polar plots in Figure S7 (R, Openair). There is a strong source of iOSs from the western side of the sampling site. Cluster analysis of air mass trajectories (Figure S8) revealed the main pathways: one involving long-distance transport, likely across the ocean, and the others from local sources such as transportation and agricultural activities. Most MTOS isomers show a similar distribution with wind speed and direction, except for MTOS_1.

All MTOS and MGOS species show a general increasing trend with temperature throughout the day and at night (Figure 3). The concentrations increased significantly at night when the temperature was from 8 to 11 °C, mainly because these temperatures are associated with a period of high pollution (PM_2.5_, SO_4_^2–^, NO) from 22nd June to 24th June, which led to a significant increase in concentrations. However, the concentrations of each iOS isomer at all temperatures during the night are generally higher than those seen during the daytime (Figure 3). This may be because, as the nocturnal boundary layer falls into the night, the concentration of all pollutants is significantly higher than during the day.

**Figure 3:**
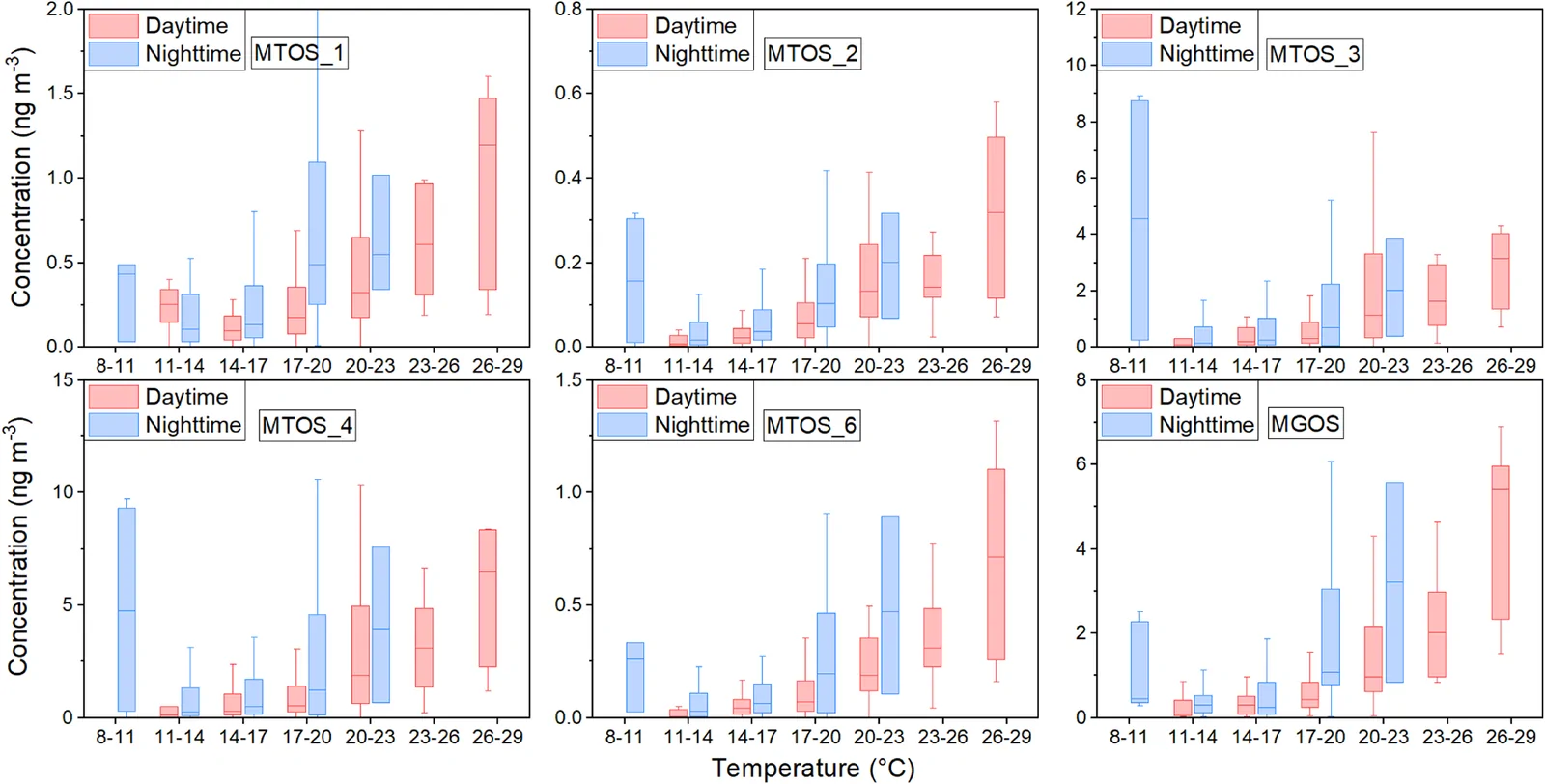
Box plots showing the relationship between [iOS] (ng m^–3^) and temperature (°C).

In previous studies, the correlation between OSs and ozone and sulfate has been seen.^15,24,48^ Bryant et al. showed a stronger correlation between iOS species with the product of ozone and particulate sulfate in Beijing, highlighting the role of both local photochemistry and particulate sulfate in iOS formation.^27^ Yang et al. showed that higher urban iOS events were associated with enhanced photochemical processes and elevated particulate sulfate levels in urban areas.^49^

During the daytime, except for MTOS_1, the concentration of other iOSs was also found to be positively correlated with the product of [O_3_].[SO_4_^2^] in Manchester (*R* = 0.556–0.690, *P* < 0.001) (Figure S9), indicating the importance of photochemistry in iOSs production. For MTOS_2, MTOS_3, and MTOS_4, we found that correlations between iOS species and [O_3_]·[SO_4_^2–^] (*r* = 0.639–0.690, *P* < 0.001) were stronger than individual correlations with either­[O_3_] (*r* = 0.556–0.643, *P* < 0.001) or [SO_4_^2–^] (*r* = 0.610–0.671, *P* < 0.001), respectively (Figure S9). The product of [SO_4_^2–^]·[O_3_] correlates better, suggesting that the formation of iOSs is driven by heterogeneous photochemical processes. This is also shown to be the case in Yang et al. for Shanghai and Liu et al. for Guangzhou.^49,50^

## 5 Conclusion

The work presented highlights the development and application of a new high-throughput isocratic elution hydrophilic interaction liquid chromatography (HILIC) method to measure isoprene-derived organosulfate tracer species (iOS) in the atmosphere. The implementation of isocratic HILIC methods provides improved analysis time and reduced solvent usage. The application of this new method for the analysis of 336 ambient samples resulted in a total savings of 61 h and 1.68 L of organic solvents, therefore offering a more sustainable, lower-cost, and time-effective approach for measuring iOSs, while ensuring accurate measurement of target compounds.

The method was applied to ambient PM_2.5_ filters collected at the Manchester Air Quality Supersite (MAQS) in the summer of 2021, which is influenced by both biogenic and anthropogenic emissions. A targeted analysis of iOS was undertaken, with tracers quantified using authentic standards. Quantified 2-methyltetrol OS (MTOS) isomer (5 isomers: C_6_H_12_O_7_S) concentrations averaged 0.36, 0.11, 1.25, 2.12, and 0.17 ng m^–3^, respectively, and the average 2-methylglyceric acid OS (MGOS: C_4_H_8_O_7_S) concentration was 1.07 ng m^–3^.

## Supplementary Material



## Notes

Supplementary analytical method validation (calibration curves, LODs, LOQs, RSDs, and matrix effects), additional meteorological and atmospheric measurements, proposed reaction pathways and chromatographic assignments, supplementary concentration, correlation, trajectory, and polar plot analyses, and summary tables describing analytical performance and campaign statistics (PDF)
