# Supplementary material for: Understanding the Temporal Evolution of Isoprene Organosulfates Using a Novel Hydrophilic Interaction Chromatography Method
Source: ACS EST Air. 2026 Jul 7;3(8):1957–65. doi: 10.1021/acsestair.5c00460 (PMC13488277; doi:10.1021/acsestair.5c00460)
Supplement: Supplementary file 1 [file ea5c00460_si_001.pdf]

Understanding the temporal evolution of isoprene organosulfates using a novel hydrophilic interaction chromatography method

Ping Liu[1,2], Daniel J. Bryant[2*], Carys Williams[2], Rhianna L. Evans[2], James R. Hopkins[2,3], Sri H. Budisulistiorini[2], Michael Flynn[4], Nathan A. I. Watson[4], James D. Allan[4,5], W. Joe F. Acton[6], William. J. Bloss[6], Connor Prior[7Ω], William Unsworth[7], Xiang Ding[1,8], Andrew R. Rickard[2,3], Jacqueline F. Hamilton[2,3*]

[1]State Key Laboratory of Advanced Environmental Technology, Guangzhou Institute of Geochemistry, Chinese Academy of Sciences, Guangzhou, 510640, China

[2]Wolfson Atmospheric Chemistry Laboratories, Department of Chemistry, University of York, York YO10 5DD, U.K

[3]National Centre for Atmospheric Science, University of York, Heslington, York, YO10 5DD, 5 UK

[4]Department of Earth and Environmental Sciences, University of Manchester, Manchester, M13 9PL, UK

[5]National Centre for Atmospheric Science, University of Manchester, Manchester, M13 9PL, UK

[6]School of Geography Earth and Environmental Science, University of Birmingham, Edgbaston, Birmingham, B15 2TT, UK

[7]Department of Chemistry, University of York, Heslington, York, YO10 5DD, UK

[8]Guangdong-Hong Kong-Macao Joint Laboratory for Environmental Pollution and Control, Guangzhou Institute of Geochemistry, Chinese Academy of Science, Guangzhou, 510640, China

[Ω]Now at School of Chemistry, University of Leeds, Leeds, LS2 9JT

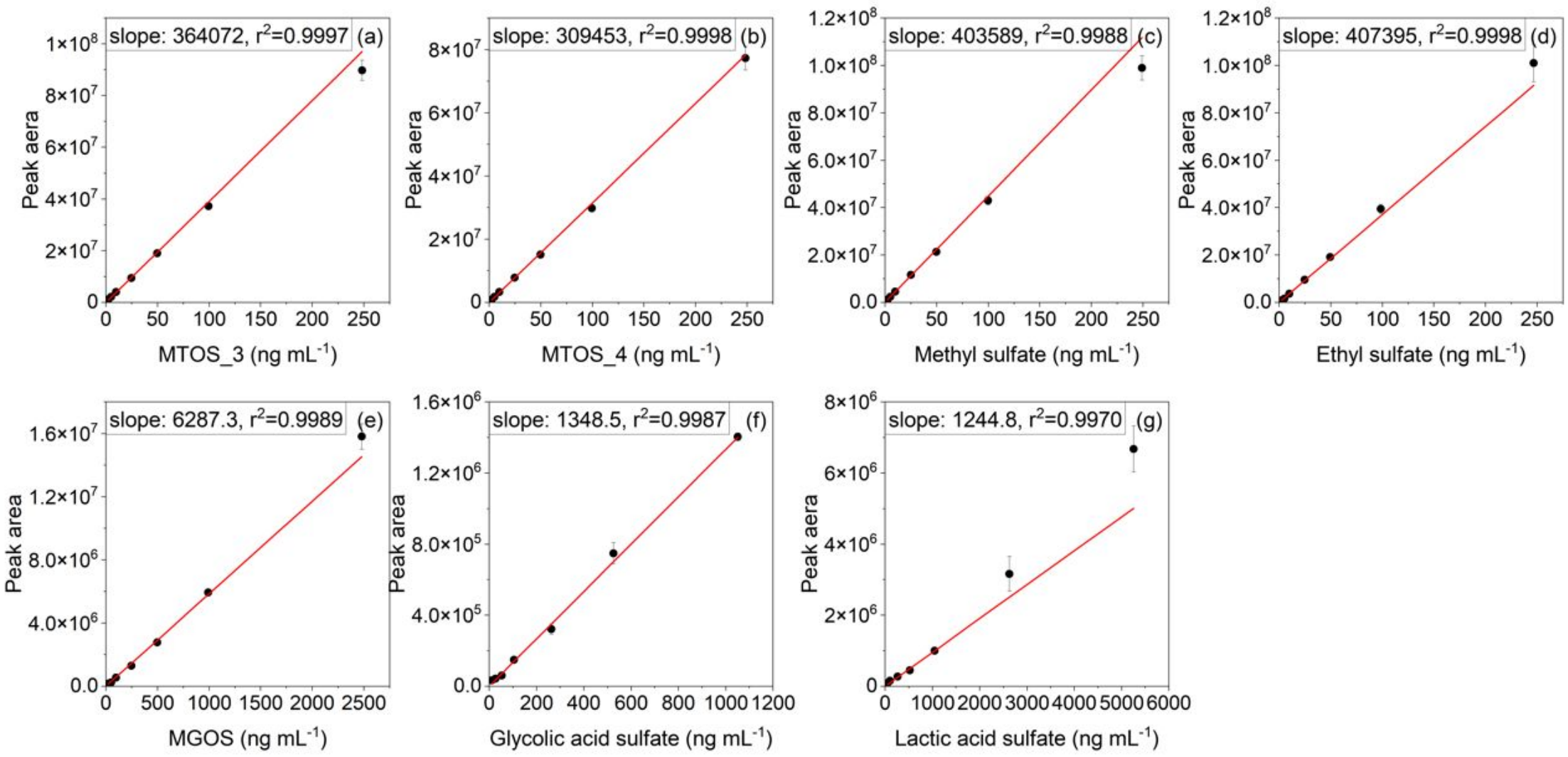

Figure S1. Correlations between peak area and concentration of standards, r^2 is the correlation coefficient.

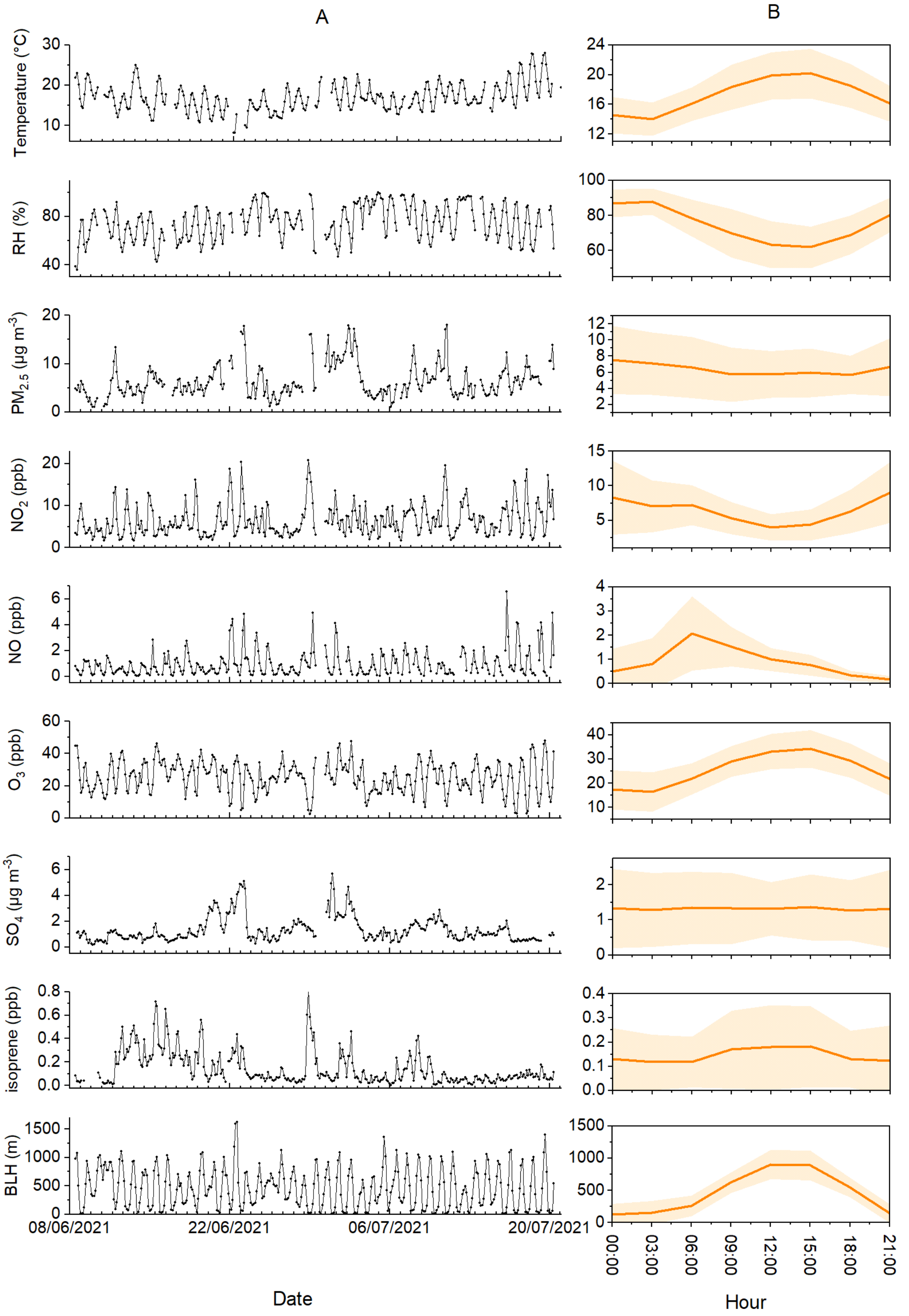

Figure S2. Time series and diel variations (mean and 95% confidence interval in mean) of temperature (℃), relative humidity (RH, %), $PM_{2.5}$ (μg m^{-3}), NO_2 (ppb), NO (ppb), O_3 (ppb) and SO_4 (ug m^{-3}) isoprene (ppb) concentration and BLH (boundary layer height: m) at the Manchester site between 08 Jun-20 Jul (with a statistical time of 3h).

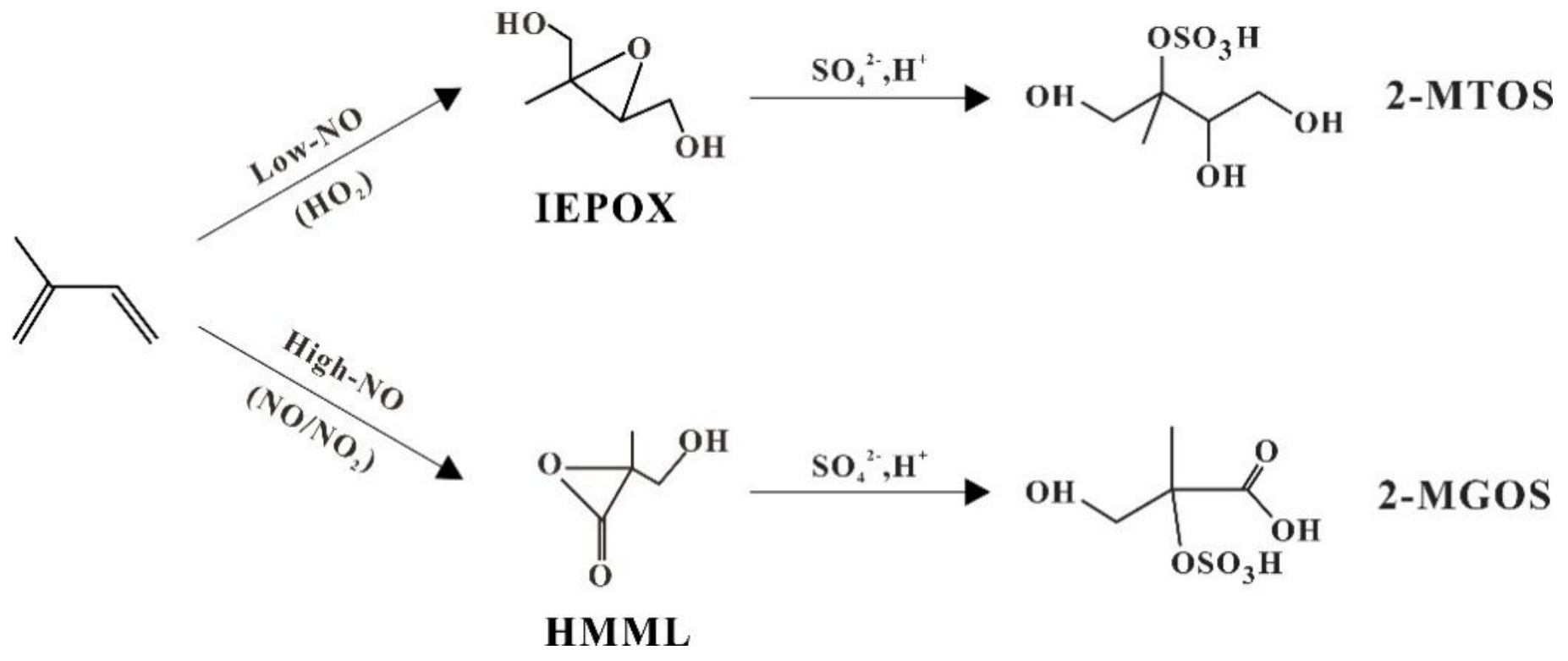

Figure S3. Simplified reaction scheme for isoprene to form organosulfates (iOS) via sulfate epoxide pathway (low NO and high NO pathways).

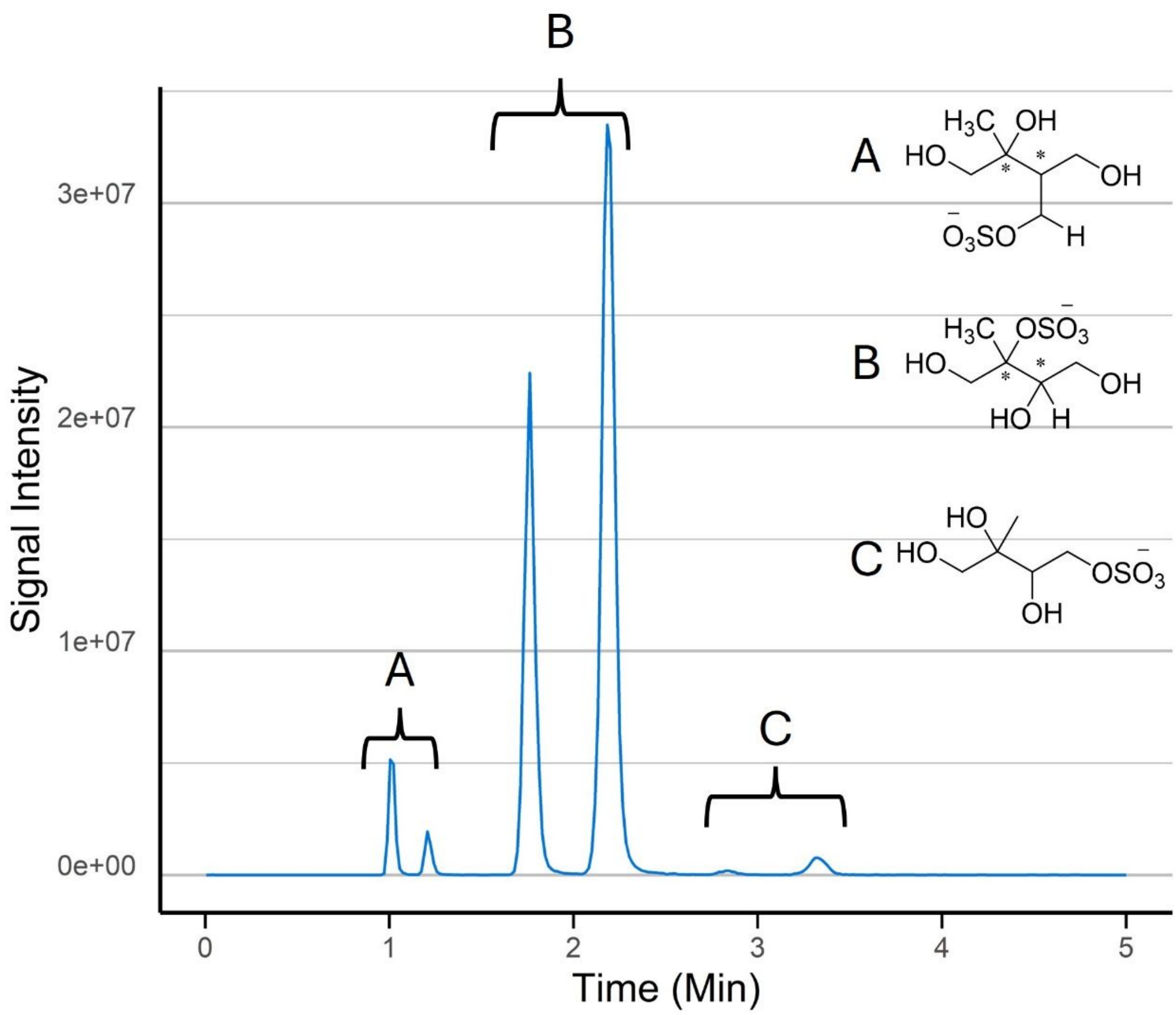

Figure S4. Extracted ion chromatogram for the MTOS with each isomer, with proposed structures on the right.

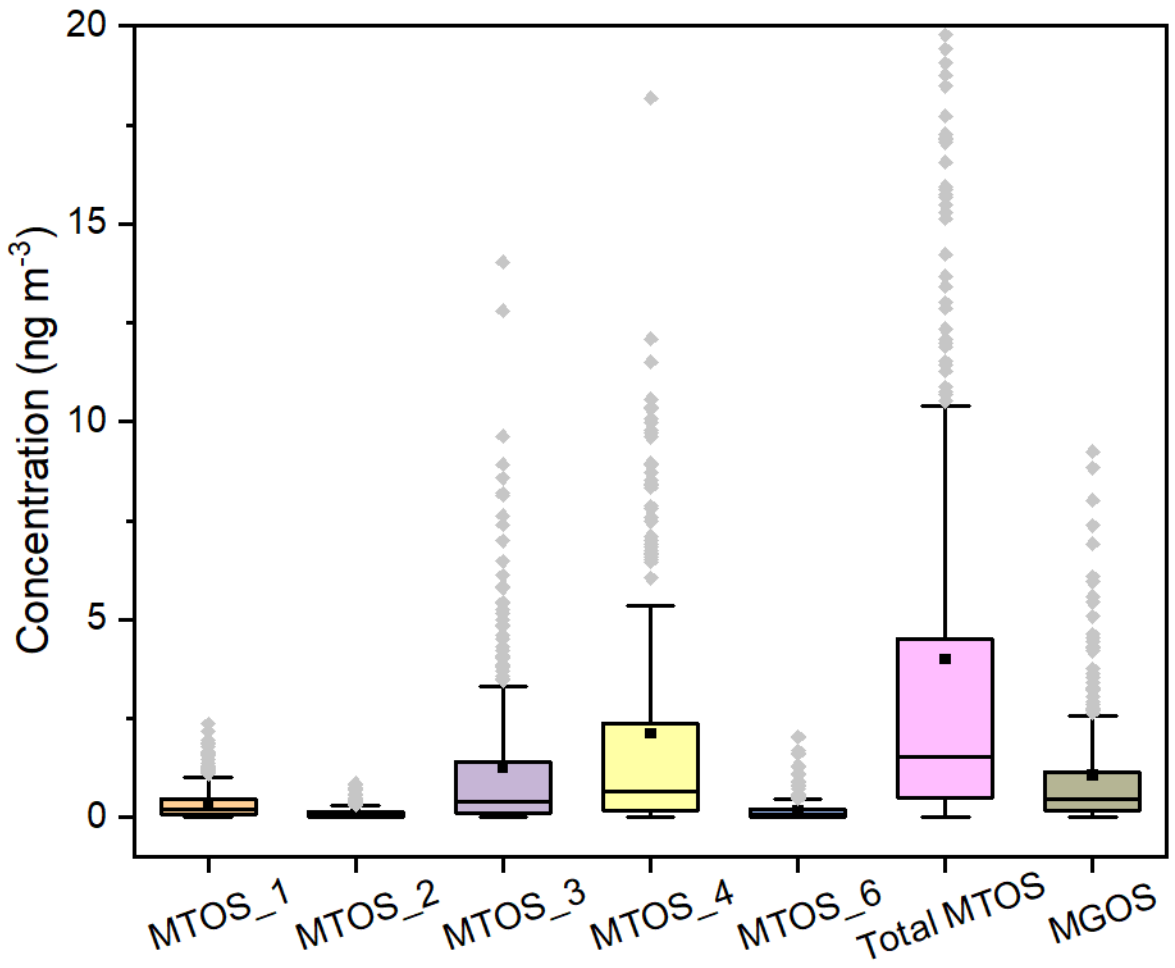

Figure S5. Concentration of MTOS isomers (ng m^{-3}) and MGOS (ng m^{-3}) at the Manchester site between 08 Jun-20 Jul.

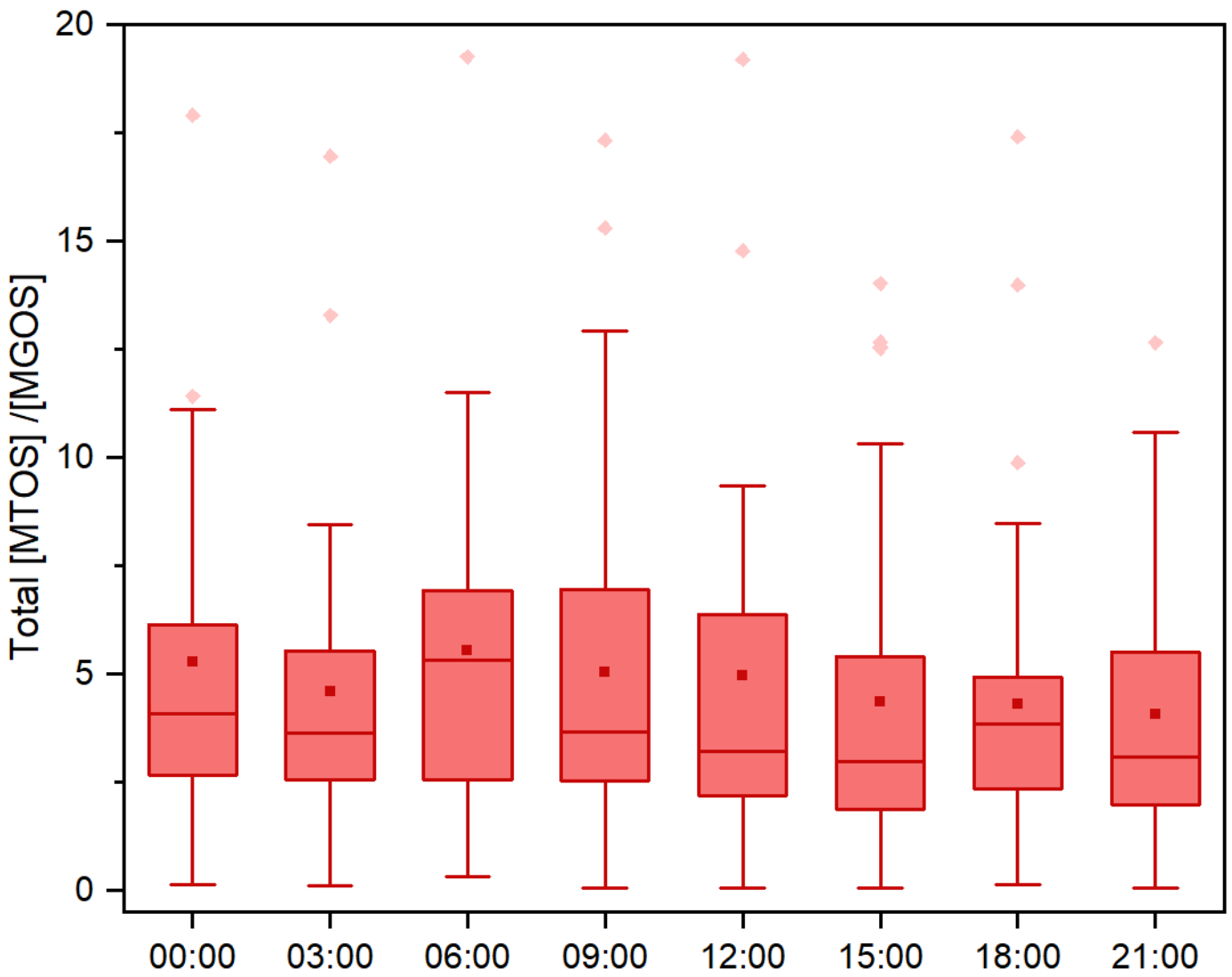

Figure S6. Diurnal variations of toatal [MTOS] vs. [MGOS] at the Manchester site between 08 Jun-20 Jul.

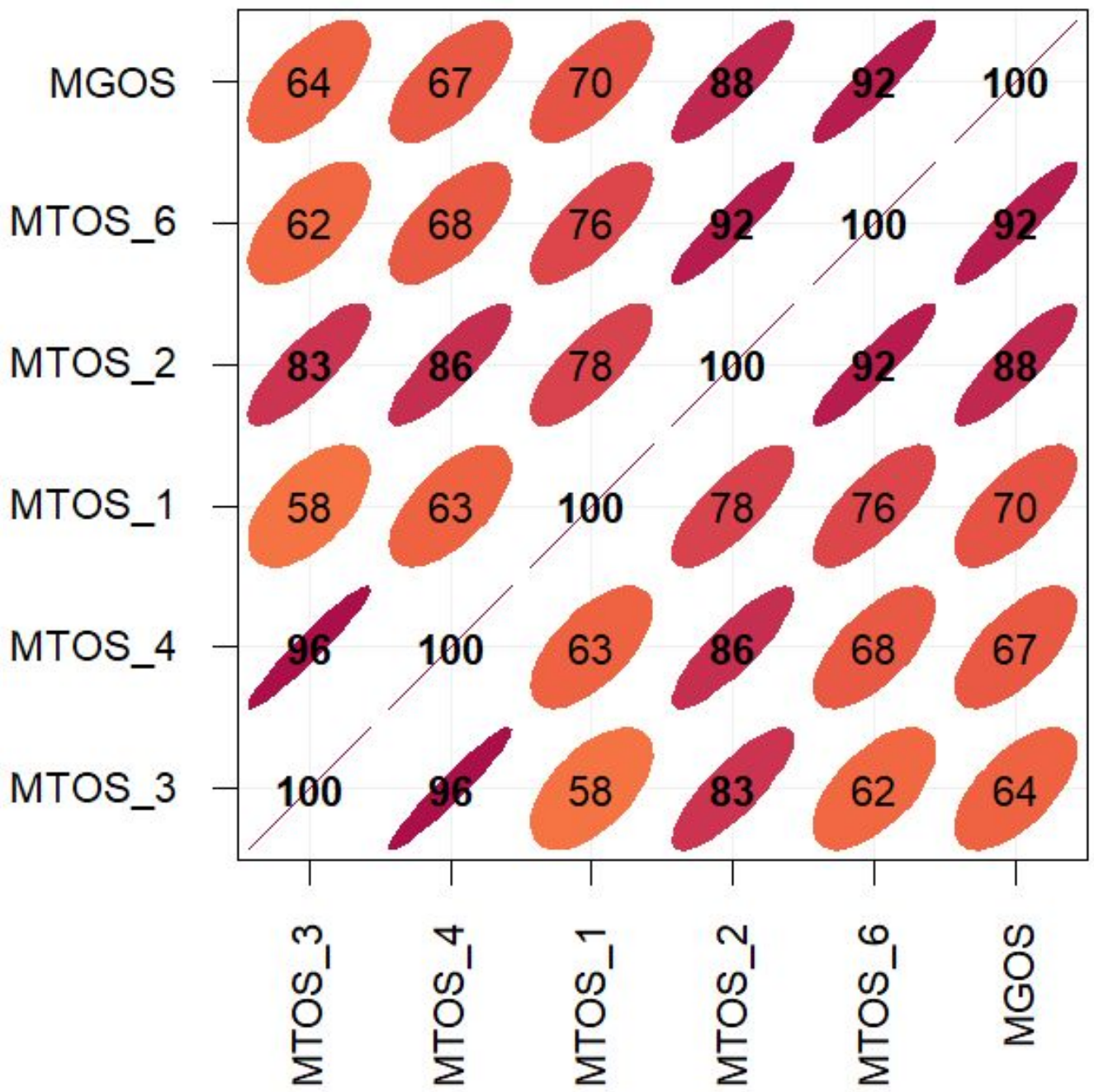

Figure S7. Correlation plot (R, Corrplot, openair) between iOS species during the campaign. The number represents the Pearson coefficient (R) between the two species, i.e., 96 is an R of 0.96. More elongated circles represent higher correlations, while darker red circles represent higher positive correlations.

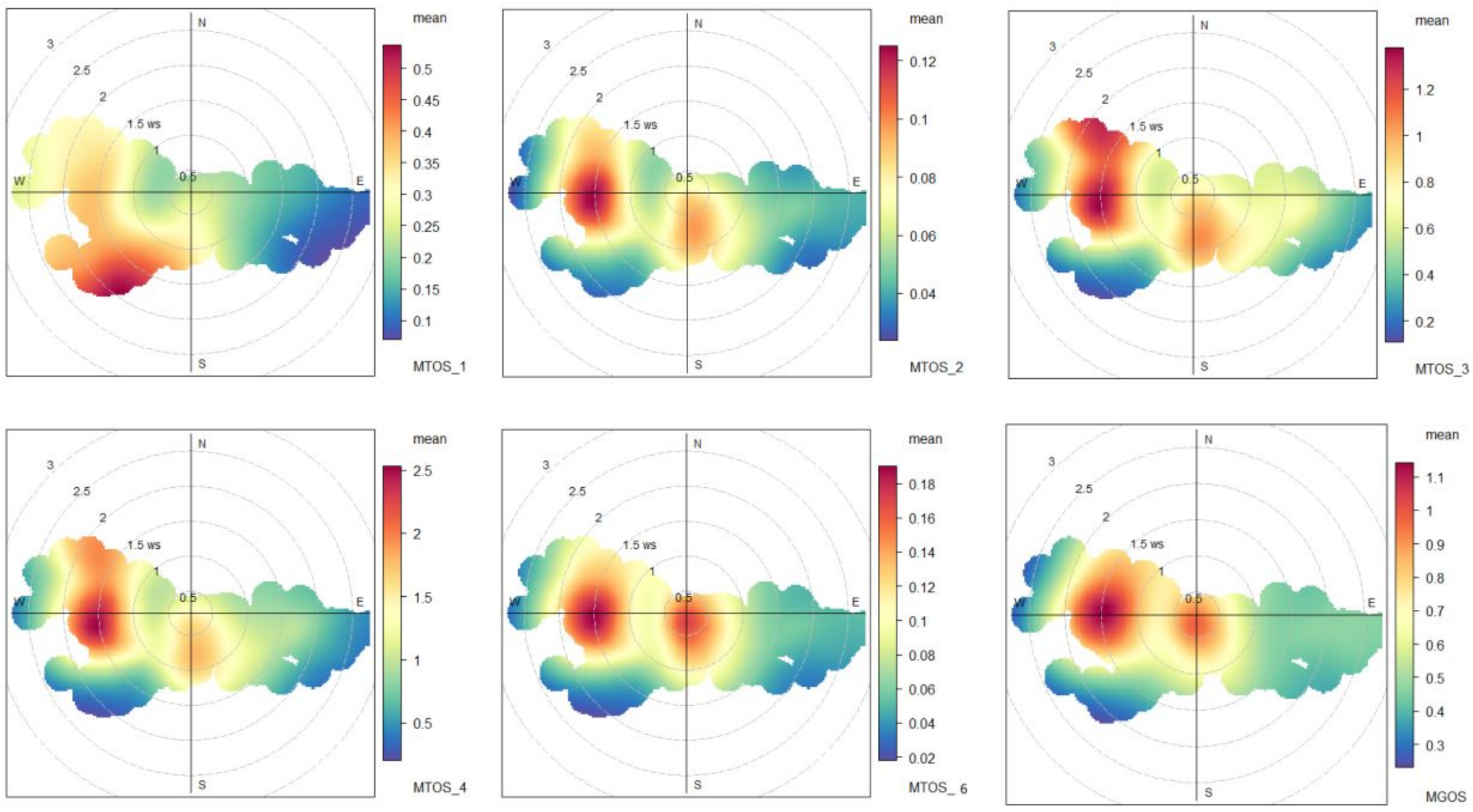

Figure S8. Pollution polar plot (OpenAir) of iOSs measured at the Manchester site between 08 Jun-20 Jul, highlighting the wind conditions under which the highest concentrations of iOSs occur.

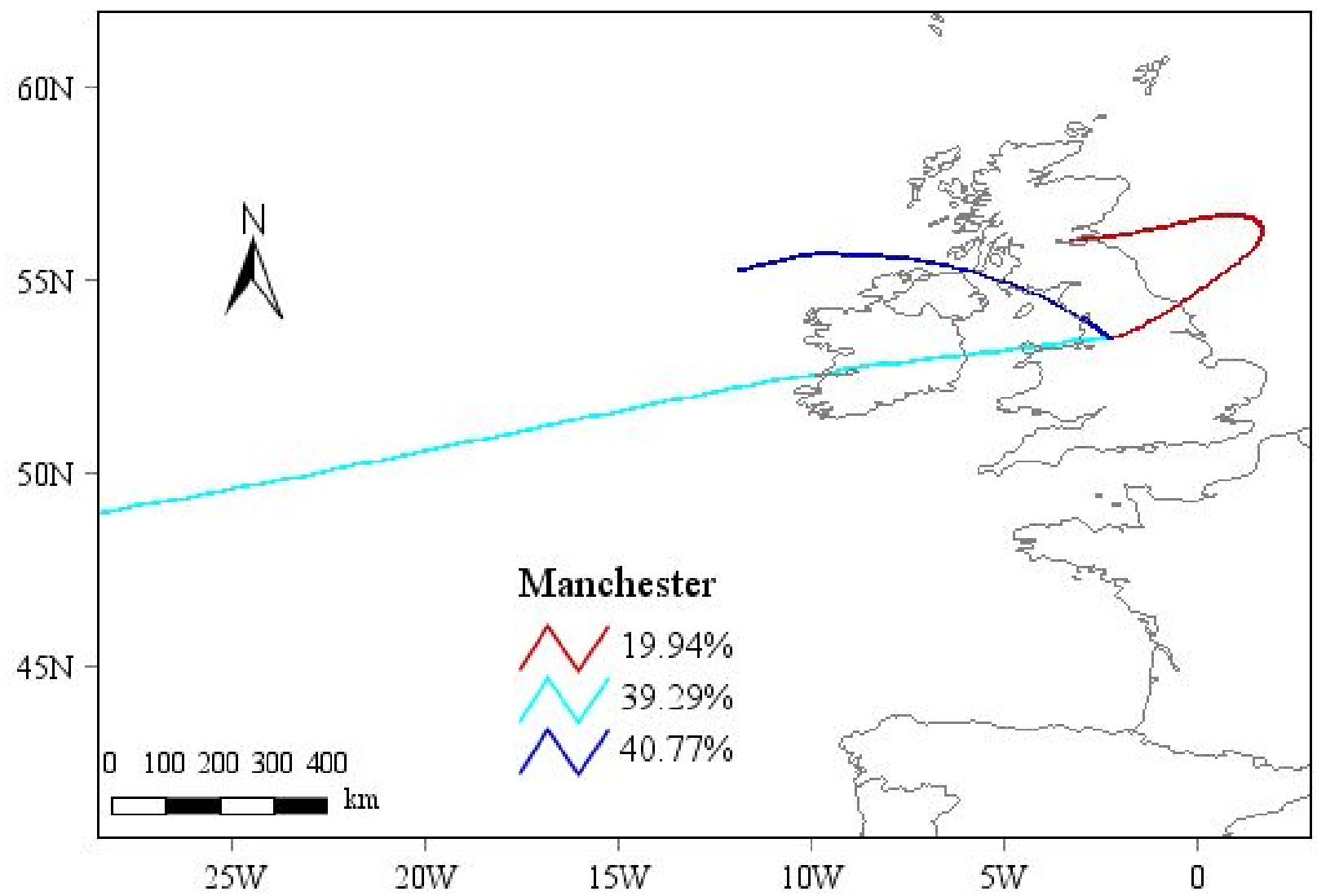

Figure S9. The air mass trajectories for the main clusters at the Manchester site between 08 Jun-20 Jul using HYSPLIT. The Calculated altitude is 100 m, the trajectory calculated time is 72 h, and the time resolution is 4 h.

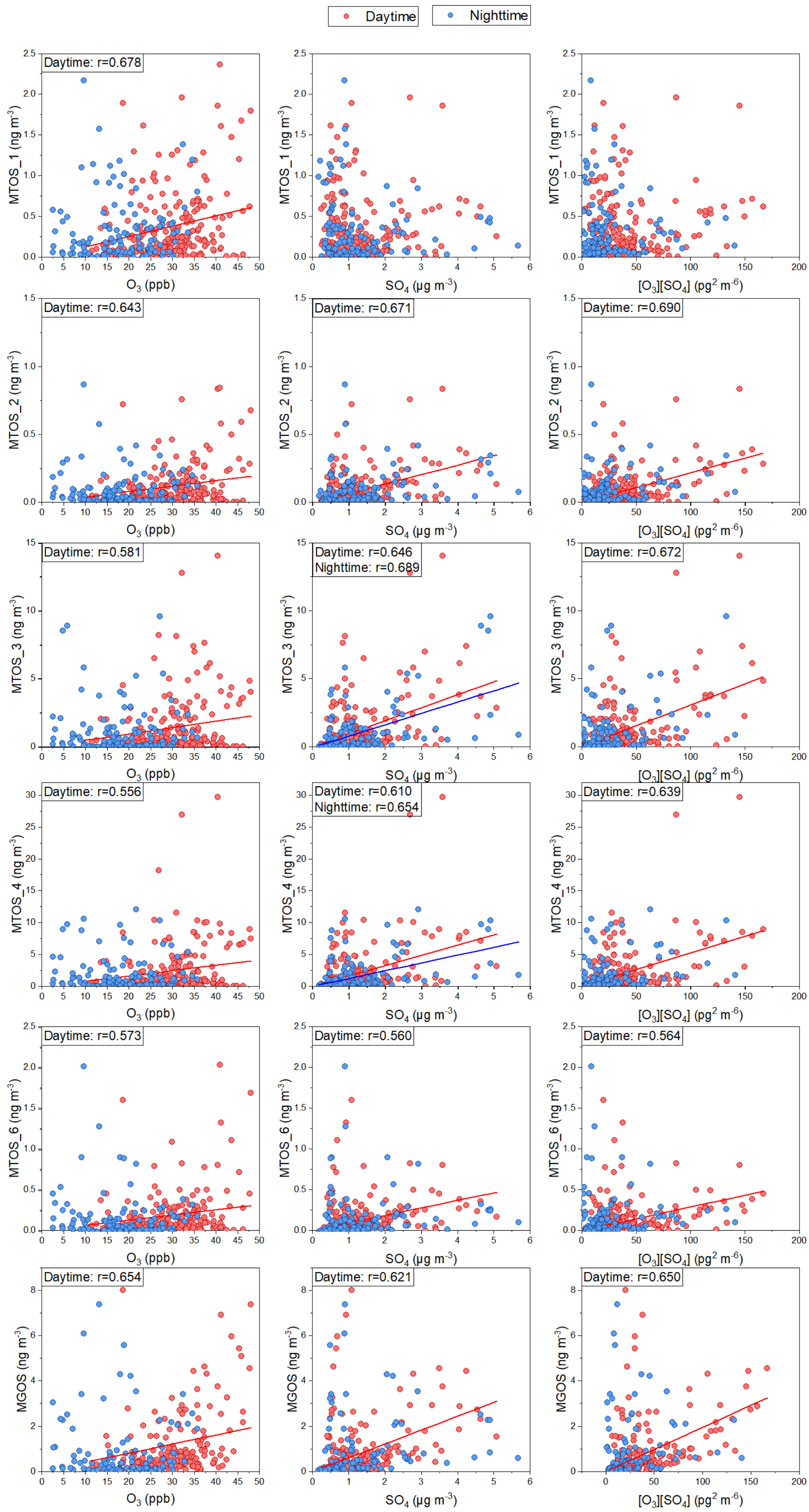

Figure S10. Correlation between MTOS and MGOS concentration and SO_4^{2-}, O_3, and $[O_3]$ [SO_4^{2-}

Table S1. The LODs: limits of detections (μg mL^{-1}), LOQs: limit of quantification (ng m^{-3}) and the RSD: relative standard deviation (%) of MS method.

**Compound**	**SD (ug mL^{-1})**	**LODs (ug mL^{-1})**	**LOQs (ng m^{-3})**	**RSD(%)**
MTOS_3	0.0077	0.005	0.095	3.75
MTOS_4	0.0067	0.004	0.084	0.12
Methyl sulfate	0.0074	0.009	0.171	4.23
Ethyl sulfate	0.0065	0.004	0.086	4.87
MGOS	0.0025	0.007	0.126	6.48
GAS	0.0058	0.017	0.309	6.12
LAS	0.0084	0.025	0.449	5.99

Table S2. Matrix effect average, maximum, minimum, and standard deviation (SD) values.

**Compound**	**Mean (%)**	**Max (%)**	**Min (%)**	**SD (%)**
MTOS_3	47.30	73.68	26.77	14.22
MTOS_4	42.23	77.15	12.22	18.68
Methyl sulfate	72.54	99.96	37.41	16.73
Ethyl sulfate	78.86	107.36	56.49	12.75

Table S3. Matrix effect of different compounds.

Date	$PM_{2.5}$	MTOS_3	MTOS_4	Methyl sulfate	Ethyl sulfate
08 Jun. FBH	/	73.69	70.08	90.92	90.18
09 Jun. 01:30	6.35	38.70	39.22	61.81	67.94
15 Jun. 07:30	8.35	55.84	53.53	61.33	84.25
19 Jun. 01:30	6.36	72.26	77.15	99.97	107.36
23 Jun. 22:30	2.81	60.03	56.11	65.22	86.33
27 Jun. 10:30	4.07	48.87	45.40	68.04	78.63
01 Jul. 13:30	11.57	37.73	27.11	37.41	75.00
06 Jul. 19:30	/	31.07	37.19	68.71	56.50
15 Jul. 16:30	7.43	36.62	27.55	59.38	62.27
20 Jul. 10:30	8.87	27.24	13.46	65.71	59.15
16 Jul. 01:30	8.50	49.14	36.15	53.18	67.11
16 Jul. 04:30	8.99	68.08	68.36	97.55	95.22
16 Jul. 07:30	12.33	40.75	22.21	59.80	77.98
16 Jul. 13:30	5.23	44.59	43.19	90.21	89.05
16 Jul. 16:30	3.57	26.78	12.22	82.66	79.60
16 Jul. 19:30	4.03	42.53	32.95	83.85	86.18
16 Jul. 22:30	6.18	50.33	56.17	93.31	82.86

Table S4. Pollutant average, maximum, minimum, median and standard deviation (SD) values at the Manchester site (with a statistical time of 3h).

	**Mean**	**Max**	**Min**	**Median**	**SD (%)**
Temp (℃)	17.09	28.00	8.11	16.79	3.49
RH (%)	75.26	99.71	35.94	75.86	14.37
$PM_{2.5}$ (ug m^{-3})	6.41	17.96	1.01	5.49	3.50
NO_2 (ppb)	6.45	20.81	1.67	5.32	3.80
NO (ppb)	0.95	6.58	BD	0.67	1.02
O_3 (ppb)	25.42	48.02	2.52	25.84	9.71
SO_4 (ug m^{-3})	1.32	5.67	0.17	1.03	0.98
isoprene (ppb)	0.144	0.810	0.004	0.083	0.143
BLH (m)	457.32	1624.2	0	426.55	356.71
MTOS_1 (ng m^{-3})	0.36	2.36	BD	0.19	0.42
MTOS_2 (ng m^{-3})	0.11	0.87	BD	0.05	0.15
MTOS_3 (ng m^{-3})	1.25	14.03	BD	0.39	1.99
MTOS_4 (ng m^{-3})	2.12	29.73	BD	0.67	3.49
MTOS_6 (ng m^{-3})	0.17	2.03	BD	0.07	0.29
Total MTOS (ng m^{-3})	3.99	47.26	BD	1.53	6.04
MGOS (ng m^{-3})	1.07	9.24	BD	0.46	1.56

Note: BD= below detection.
